# Genome Characterization, Comparison and Phylogenetic Analysis of Complete Mitochondrial Genome of *Evolvulus alsinoides* Reveals Highly Rearranged Gene Order in *Solanales*

**DOI:** 10.3390/life11080769

**Published:** 2021-07-30

**Authors:** Pattayampadam Ramakrishnan Shidhi, Vadakkemukadiyil Chellappan Biju, Sasi Anu, Chandrasekharan Laila Vipin, Kumar Raveendran Deelip, Sukumaran Nair Achuthsankar

**Affiliations:** 1 Department of Computational Biology and Bioinformatics, University of Kerala, Thiruvananthapuram 695581, India; bijuvcdcb@keralauniversity.ac.in (V.C.B.); anu24sasi@gmail.com (S.A.); vipincl1993@gmail.com (C.L.V.); achuthsankar@keralauniversity.ac.in (S.N.A.); 2 Campus Computing Facility (CCF) at the Central Laboratory for Instrumentation and Facilitation, University of Kerala, Thiruvananthapuram 695581, India; deelip@keralauniversity.ac.in

**Keywords:** *E. alsinoides*, Convolvulaceae, *Solanales*, *mitogenome*, protein-coding genes

## Abstract

Mitogenome sequencing provides an understanding of the evolutionary mechanism of mitogenome formation, mechanisms driving plant gene order, genome structure, and migration sequences. Data on the mitochondrial genome for family Convolvulaceae members is lacking. *E. alsinoides*, also known as shankhpushpi, is an important medicinal plant under the family Convolvulaceae, widely used in the Ayurvedic system of medicine. We identified the mitogenome of *E. alsinoides* using the Illumina mate-pair sequencing platform, and annotated using bioinformatics approaches in the present study. The mitogenome of *E. alsinoides* was 344184 bp in length and comprised 46 unique coding genes, including 31 protein-coding genes (PCGs), 12 tRNA genes, and 3 rRNA genes. The secondary structure of tRNAs shows that all the tRNAs can be folded into canonical clover-leaf secondary structures, except three *trnW, trnG*, and *trnC*. Measurement of the skewness of the nucleotide composition showed that the AT and GC skew is positive, indicating higher A’s and G’s in the mitogenome of *E. alsinoides*. The Ka/Ks ratios of 11 protein-coding genes (*atp1*, *ccmC*, *cob*, *cox1*, *rps19*, *rps12*, *nad3*, *nad9*, *atp9*, *rpl5*, *nad4L*) were <1, indicating that these genes were under purifying selection. Synteny and gene order analysis were performed to identify homologous genes among the related species. Synteny blocks representing nine genes (*nad9*, *nad2*, *ccmFc*, *nad1*, *nad4*, *nad5*, *matR*, *cox1*, nad7) were observed in all the species of *Solanales*. Gene order comparison showed that a high level of gene rearrangement has occurred among all the species of *Solanales*. The mitogenome data obtained in the present study could be used as the *Convolvulaceae* family representative for future studies, as there is no complex taxonomic history associated with this plant.

## 1. Introduction

Plastid, mitochondrial, and nuclear genomes constitute the total genome of a plant cell. There are around 1000 copies of chloroplast DNA, 100 copies of mitochondrial DNA (mtDNA), and 2 copies of nuclear DNA in a typical *Arabidopsis* leaf cell [1]. In comparison to the conserved chloroplast genome, mitogenomes are unique to each plant, and their analysis will help understand the evolutionary patterns better. Additionally, since mitogenomes contain phylogenetic details, they can reconstruct robust phylogenies in various taxa [2,3]. The mitogenomes show remarkable variation among land plants, and their size is usually larger than plastid genomes. Despite their relatively large size, mitogenomes contain fewer genes than their plastid counterparts [4,5]. Plant mitogenomes are remarkably different from animal mitogenomes [6]. It shows a distinctive 200-fold size divergence, ranging from 66kb in *V. scurruloideum* to 11.3 Mb in *S. conica*, due to the expansion primarily from repeat sequence, intronic regions, and the incorporation of plastid and nuclear DNA through intracellular gene transfer (IGT) or foreign mtDNA via horizontal gene transfer (HGT) [7,8]. The accumulation of these repeat sequences in vascular plants causes active recombination and serves as a source of rearrangements, leading to the generation of alternative structure, even within a single individual [9]. These rearrangements are facilitated by homologs of bacterial DNA repair proteins, such as RecA and MutS [10]. Due to rearrangements, plant mtDNA is referred to as multipartite, interconverting between a master circle and subgenomic circles or minicircles [11]. Plant mtDNA evolves much more slowly in sequence than animal mtDNA does, considering its structural heterogeneity and gene sequences have meager base substitution rates. This could be because of an active homologous recombination mechanism, part of the repair system that potentially copy-corrects mutations by gene conversion [6]. Also, plant mitogenomes are not restricted to a single origin of replication. In *Arabidopsis thaliana* and *Nicotiana tabacum*, two organellar DNA polymerases are dual-targeted to both mitochondria and plastids, termed Pol1A and Pol1B [12,13].

An essential element of mitochondrial genetics is RNA editing, which is implicated in various plant developmental processes, including organelle biogenesis, environmental adaptability, and signal transduction [14,15]. RNA editing involves converting cytosine to uracil, forming a corrective mechanism for overcoming potentially deleterious mutations [16,17]. The gene family that is responsible for the RNA edits in land plants is the nuclear-encoded pentatricopeptide repeat (PPR) [18]. The nucleotide changes that are created by the editing events mainly occur at the first and second codon positions, leading to amino acid changes that appear to be conserved along with the evolution. These events are suggested to provide a control point at the initiation of translation [19]. Genome sequence data help to understand the degree of variation in editing between recently diverged lineages and the effect of interspecific hybridization on the patterns of editing. Such variations contribute to adaptation and population-level divergence [20]. Even though there is a high gene content conservation between close relatives, their gene order may differ significantly due to the frequent DNA repair via homologous recombination (HR) and non-homologous end-joining (NHEJ). Also, in some cases, there exists a remarkable difference in the shared sequence between closely related plants. So, sufficient sequence data are needed for the efficient comparative study of different phylogenetic groups of plants [21]. More than 100 angiosperm mitogenomes have been sequenced (http://www.ncbi.nlm.nih.gov/genome/organelle/, accessed on 9 October 2020), which show a remarkable variation in genome size, content, and structure. However, in angiosperms, sequencing and analysis of mitogenomes are more difficult, due to variations in the non-coding regions and low conservation across species [22]. With recent advances in DNA sequencing technology and efficient bioinformatics tools, there has been rapid growth in plant mitogenome projects and high-quality mitogenome assemblies. *A. thaliana* ecotype C24 mtDNA of 367 kb size was the first angiosperm mitogenome that was entirely sequenced. It codes for 32 proteins, 3rRNAs, and 22tRNAs [23,24]. Recent research has leveraged the growing availability of paired-end libraries and mate-pair libraries to complete mitogenome assemblies.

The mitochondrial genome of no species under the Convolvulaceae family has been sequenced and characterized yet. The family Convolvulaceae includes 85 genera and about 2800 species [25]. The mentioned family has several plants with medicinal properties. *E. alsinoides* is a short, hairy, procumbent, diffuse perennial herb with a small woody branched rootstalk, and is native to South America and widely distributed in tropical and subtropical regions of the world [26,27]. It is included in various traditional medicines, especially in ayurvedic formulations, such as Brahma rasayana, Jeevanyadi ghrita, Brahmi ghrita, Vachadi ghrita, Naladi ghrita, and Agastya rasayana [28]. Root and stem extracts of the plant are used to treat dysentery and depression, while the leaves are used to treat asthma and mental disturbances [29]. The various phytochemicals that are present in the plant possess pharmacological activities such as anxiolytic, anti-amnesic, tranquilizing, anti-depressant, anti-stress, antioxidant, antidiabetic, and anti-ulcer properties [30]. Since this plant has no complex taxonomic history, the mitogenome of this plant can be used as the first representative from the family *Convolvulaceae* [27,31]. So, the objective of the present study is to sequence and assemble the complete mitogenome of *E. alsinoides*. The complete genome was annotated to analyze its gene content, genome composition, repetitive sequences, RNA editing sites, synonymous (Ks) and nonsynonymous (Ka) substitution rates, codon usage profile, phylogenetic analysis, gene arrangement, synteny analysis, and a complete comparison with all the available mitogenome of *Solanales* was performed. In the future, this will be a valuable resource for further studies on mitogenome in the family *Convolvulaceae*, as it is the first sequenced mitogenome in the family.

## 2. Materials and Methods

### 2.1. Plant Material, Library Preparation and Genome Sequencing

Fresh leaves of a single *E. alsinoides* plant (specimen voucher no. KUBH9910) were collected from Kerala, southern state in India. Total genomic DNA was isolated from the fresh leaves using CTAB (cetyl trimethyl ammonium bromide) method [32]. The sample’s purity was evaluated using nanodrop (Nanodrop Technologies, Wilmington, DE, US), and the sample was quantified using the Qubit system (Thermo Fisher Scientific, Waltham, MA, USA). Then, 1 µg high-quality genomic DNA was used for mate-pair library preparation. Libraries were constructed using the Illumina Nextera mate-pair library prep kit (FC-132-1001) (Illumina, San Diego, CA, USA) using a standard protocol. The library quality and quantity were assessed using TapeStation 4200 (Agilent Technologies, Santa Clara, CA, USA). Illumina mate-pair library was prepared with an average insert size of 10 kb and was sequenced using an Illumina HiSeqX—2 × 150 bp PE platform (Illumina, San Diego, CA, USA). The raw reads used for the study are available in the NCBI Sequence Read Archive (SRA) under the SRA accession number: PRJNA551197.

### 2.2. Mitochondrial Genome Assembly

The raw reads quality check was performed using FastQC (v0.10.0) (available online: http://www.bioinformatics.babraham.ac.uk/projects/fastqc/, accessed on 28 January 2020). Preceding to the genome assembly, reads containing adaptor sequences were removed using Cutadapt v1.8 [33], and the quality trimming of the reads was performed using Sickle v1.33 (available online: https://github.com/najoshi/sickle, accessed on 9 February 2020). The mate-pair high-quality reads were mapped to the previously published mitogenomes of land plants from NCBI organelle genome resources to filter the mitochondrial reads using BWA v0.7.12-r103 [34]. The filtered reads were de novo assembled using SPAdes-3.13.0 [35]. The resulting pre-assembled contigs were extended and scaffolded using SSPACE v2.0 [36]. The quality of assembly was evaluated using QUAST [37]. The initial assembly generated 4 contigs; three small gaps found in the intergenic region with sizes 4, 7, 5, respectively, were filled with reference genomes of *E. alsinoides* (PRJNA551197) and closely related species to get the complete genome. Gap filling was performed after comparing the mitogenome of *E. alsinoides* and 9 species of the order *Solanales* using BLAST analysis [38] (Supplementary Table S9) [38]. The final assembly was validated by mapping the mitochondrial genome assembly with the Illumina mate-pair reads using BWA v0.7.12-r103 [34].

### 2.3. Mitochondrial Genome Annotation

Preliminary mitogenome annotation was performed using GEseq webserver [39] (available online: https://chlorobox.mpimp-golm.mpg.de/geseq.html, accessed on 25 February 2020). Few of the mitochondrial features were not properly annotated using Geseq, hence MITOS [40] (available online: http://mitos.bioinf.uni-leipzig.de/index.py, accessed on 6 March 2020) was also used to annotate the genome. A BLAST analysis was performed using the mitogenome as a query and NCBI organelle genome as a database to identify PCGs and rRNAs to validate the above prediction. The genes coordinates, start, and stop position of the genes were manually corrected. The tRNA genes were annotated using tRNAscan-SE v2.0 [41,42] (available online: http://lowelab.ucsc.edu/tRNAscan-SE/, accessed on 10 March 2020) with default settings. OrganellarGenomeDRAW [43] (OGDRAW) (available online: https://chlorobox.mpimp-golm.mpg.de/OGDraw.html, accessed on 14 March 2020) program was used to draw the circular map for the genome. The complete mitogenome sequence of *E. alsinoides* was deposited under the GenBank accession number MT081228. The skewness of the nucleotide compositions was measured according to the following formulas: AT skew [(A − T)/(A + T)] and GC skew [(G − C)/(G + C)] [44,45].

### 2.4. Repetitive Sequence Analysis

Forward, reverse, palindrome, and complementary repeats were identified using REPuter v2.74 [46] (available online: https://bibiserv.cebitec.uni-bielefeld.de/reputer/, accessed on 23 July. 2020), with minimum repeat size taken as 20 bp. Simple sequence repeats SSRs were detected using MISA [47] (available online: https://webblast.ipk-gatersleben.de/misa/, accessed on 8 August 2020). A comparison was performed with 9 species of *Solanales*. The results obtained by various programs were manually consolidated, and redundancy was removed.

### 2.5. RNA Editing Site Prediction

RNA editing site present in the PCGs of *E. alsinoides* mitogenome were predicted using PREP-mt [48] (available online http://prep.unl.edu/, accessed on 20 September 2020) and a comparison was made with the 9 selected species of *Solanales*. The cut-off value C = 0.6 was used in the process for accurate prediction.

### 2.6. Synonymous and Nonsynonymous Substitution Ratio

The 31 PCGs of *E. alsinoides* and 9 species of the order *Solanales* were aligned separately using MAFFT v7 [49] (available online: https://mafft.cbrc.jp/alignment/server/, accessed on 3 October 2020) and translated to protein sequence. Nonsynonymous (Ka) and synonymous (Ks) substitution ratio (Ka/Ks) of the mitochondrial PCGs of *E. alsinoides* were predicted using DnaSP v5.10.01 [50,51] to identify the genes that are under selection pressure. Statistical analysis of Ka/Ks ratio of 19 protein coding genes were carried out by performing one-way Anova using GraphPad prism v8.0.2. (GraphPad Software, San Diego, CA, USA). Codon usage analysis was performed using CodonW v1.4.4 (available online: http://codonw.sourceforge.net/, accessed on 19 October 2020).

### 2.7. Gene Arrangement and Synteny Analysis

Mitogenome organizations of all the species were detected by using MAUVE multiple genome alignments [52]. The gene location and arrangement were verified by the linear genome map generated by OGDRAW. The synteny analysis was performed using MAUVE 2.3.1 [53] software. CREx [54] was used for pairwise comparisons of the gene order. MLGO web server [55] (http://www.geneorder.org/server.php, accessed on 7 November 2020) was used to infer a phylogeny from gene order data.

### 2.8. Phylogenetic Analysis

For phylogenetic analysis, the following two families were included within the order *Solanales*: *Convolvulaceae* and *Solanaceae*. All the mitogenomes under the order *Solanales*, available in the NCBI organelle genome, were selected for phylogenetic analysis. A total of 11 species (Supplementary Table S1) including 8 *Solanales* (3 *Nicotiana* species, 2 *Solanum* species, *C. annuum*, *H. niger*, and *P. orientalis*), 2 *Convolvulaceae* (*I. nil* and *E. alsinoides*), and 1 *Brassicaceae* (*A. thaliana*) as the outgroup were selected for constructing the phylogenetic tree. Maximum likelihood (ML) and Bayesian inference (BI) methods were employed to construct a phylogenetic tree. The mitogenome sequences were retrieved from the NCBI database (https://www.ncbi.nlm.nih.gov/genbank/, accessed on 14 December 2020). Nineteen shared PCGs were aligned separately using MAFFT v7 [49]. Gblock v.0.19b [56] was used to discard the hypervariable regions and poorly aligned nucleotides from the alignment using default parameters. All the individual gene alignments were concatenated to generate a super gene alignment. The best partitioning scheme for ML and BI phylogenetic analysis was determined using PartitionFinder2 [57]. For an additional confirmation Modeltest-NG [58] was performed to identify the best nucleotide substitution model. The model selection was based on Akaike’s information criteria (AIC) and Bayesian information criteria (BIC) with a greedy search algorithm and branch lengths linked.

Partitioned 1_N_2_N_ and 1_N_2_N_3_RY_ sequences were used for constructing the phylogenetic tree using ML and BI methods. The ML phylogenetic tree was constructed using RAxML v8.2.12 [59] with a GTR + G + I model of evolution using 1000 bootstrap replicates. Bayesian inference (BI) was performed using MrBayes v3.2.6 [60] with the model determined by the PartitionFinder2. Bayesian analysis was executed for two million generations, with sampling done at every 100 generations. Metropolis-coupled Markov chain Monte Carlo (MCMCMC) method was used with four chains (one cold chain and three heat chains). The first 5000 sampled trees were discarded as burn-in, and the remaining 15,000 sampled trees were used to construct the consensus tree and posterior probabilities. Two independent runs were used to provide additional confirmation of convergence of posterior probability distribution. The convergence of run was assessed by the log-likelihood (InL) values and effective sample size (ESS) using Tracer v1.7.2 [61]. The ESS values above 200 were considered as good indicators of convergence [62]. The long branch length of *E. alsinoides* was analyzed by constructing the phylogenetic tree using the following different approaches: (a) after excluding long branch taxa; (b) after excluding outgroup taxa; (c) after eliminating third codon positions partitioning; (d) sampling more taxa [63,64,65,66,67].

## 3. Results and Discussion

### 3.1. Mitogenome Structure, Organization and Composition

**Figure 1:**
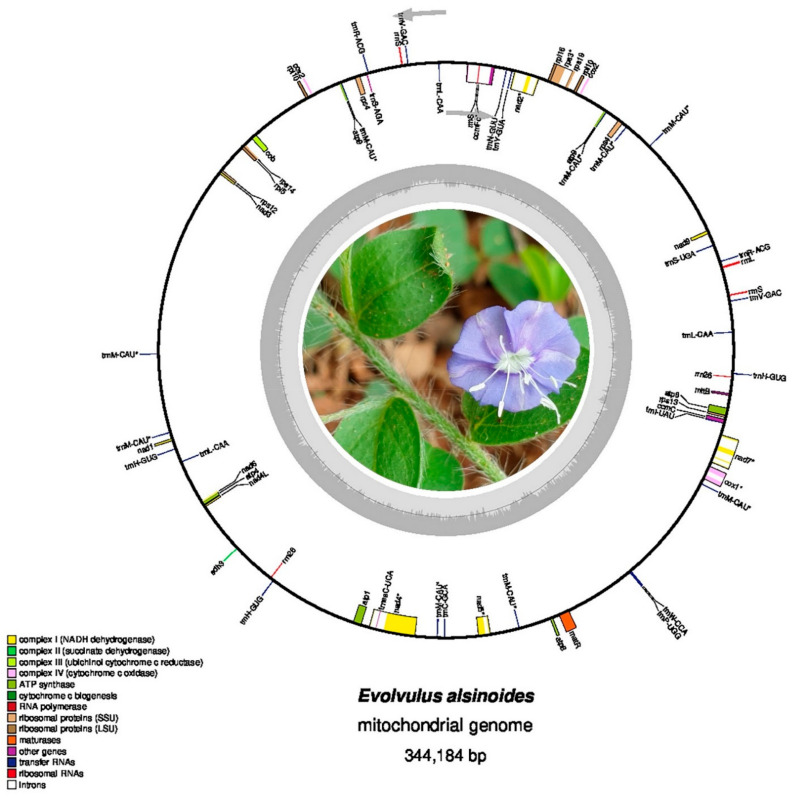
The complete Mitogenome map of *E. alsinoides*. Genes represented on the outside of the circle are transcribed counter-clockwise, whereas those inside are transcribed clockwise. The genes are represented as color bars in the circle based on their function. The inner dark grey and light grey circle indicated the GC and AT content of the mitochondrial genome.

A total of 328,334,850 raw reads were generated after the sequencing, and 133,876,340 high-quality reads were obtained after quality trimming. Out of this, 5,097,201 reads were mapped to the mitochondrial origin. The primary de novo assembly using SPAdes consisted of 3578 contigs with GC% of 39.9% and N50 of 2363 bp. Of these primary contigs, extension and scaffolding using SSPACE resulted in 756 contigs with GC% of 39.6% and N50 of 3326 (Supplementary Table S1). After the gap-filling step, the *E. alsinoides* mitochondrial genome was assembled into a single, circular molecule with a length of 344,184 bp (Figure 1). The nucleotide composition of the whole mitogenome was A: 28.28%, C: 21.73%, G: 21.81%, and T: 28.18%. The AT and GC contents of the mitogenome were found to be 56.46% and 43.54%, respectively. The AT (0.001631254) skew and GC (0.001835083) skew were positive, indicating higher A’s and G’s in the mitochondrial genome of *E. alsinoides*. The mitogenome encoded 46 unique genes, including 31 PCGs, 12 tRNA genes, and 3 rRNA genes (Table 1, Supplementary Table S2). The complete mitogenome sequence of *E. alsinoides* has been deposited in the GenBank database with the accession number MT081228.

### 3.2. Protein-Coding Genes

Among the PCGs, four PCGs (*atp9, cox2, rps4, rpl10*) were duplicated in the mitochondrial genome of *E. alsinoides*. The number of introns varyied among the PCGs. The genes *nad2, nad4, nad5, nad7, cox1, rps3*, and *ccmFc* exhibited a single intron (Table 1), *nad2* possessed two introns, and *nad7* exhibited three introns. The length of the protein-coding regions ranged from 101 to 4506 (Supplementary Table S2). The entire length of PCGs was 26,935 bp among the whole mitogenome. The nucleotide composition of the mitogenome’s protein-coding region was A: 27.54%, C: 20.68%, G: 21.28%, and T: 30.50%. The AT and GC content of the mitochondrial protein-coding genes were found to be 58.04% and 41.96%, respectively. The GC (0.014156786) skew was positive, indicating a higher G content than C, whereas the AT (−0.050853963) skew was negative, indicating a higher T content than A among PCGs of the mitochondrial genome.

### 3.3. Transfer and Ribosomal RNA Genes

A total of 12 unique tRNAs were identified in the mitogenome (Supplementary Table S3), and, among these, five tRNAs *(trnH, trnL, trnM, trnR* and *trnV*) were found in multiple copies. Nine copies of *trnM*, three copies of *trnH* and *trnL*, and two copies of *trnR* and *trnV* genes were found in the mitogenome. Three tRNAs (*trnW*, *trnG*, *trnC*) exhibited an intron in their gene. The total size of the tRNA was 1962 bp, with PCGs size ranging from 64 bp to 86 bp (Supplementary Table S3). The secondary structure of the tRNAs showed that most of the tRNAs could be folded into canonical clover-leaf secondary structures, except three (one each for *trnM*, *trnY* and *trnI)*. The total size of the rRNA genes was 724 bp, which was composed of duplicated copies of *rrn26*, triplicated copies of *rrnS*, and a single copy of *rrnL* (Table 1, Supplementary Table S2).

### 3.4. Comparative Analysis of Mitogenomes of *Solanales*

The *E. alsinoides* genome in the present study is an important resource to understand the basic genome features and gene arrangement in the family *Convolvulaceae*. A comparison of the *E. alsinoides* genome with other species of *Solanales* showed that the size of the genome varies among all the species, but the number of PCGs do not vary. *Solanum pennellii* has the highest number of PCGs, and *Ipomoea nil* has the lowest number of PCGs (Table 2). The AT and GC content in the genome range from 54 to 56% and 43 to 45%, respectively (Table 2). The *E. alsinoides* genome has the highest AT% and it has the lowest GC% among all the species (Table 2). *S. pennellii* and *Physochlaina orientalis* have negative AT and GC skew values. *I. nil* and the *Solanum lycopersicum* mitochondrial genome have positive AT skew and negative GC skew values, whereas *Hyoscyamus* *niger* has negative AT skew and positive GC skew values. Many species *(E. alsinoides*, *Nicotiana Sps*. and *C. annum*) show positive AT and GC skews in the mitochondrial genome, indicating that these genomes have higher As and Gs in their genome. The genome comparison of *E. alsinoides* with *Solanales* exhibited 19 shared genes (Supplementary Table S4).

### 3.5. Repetitive Sequence Analysis

**Figure 2:**
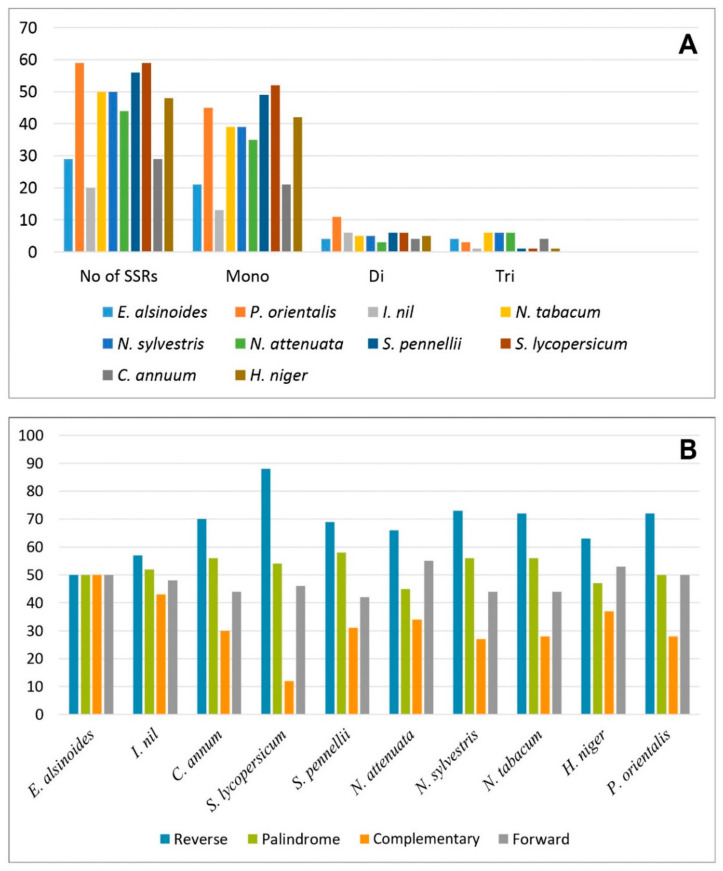
Repetitive sequences of all the species. (**A**) The total number of SSR repeats and different repeats present in each species was represented in the graph. The *x*-axis represents the number and type of SSR and *y*-axis represents the number of repeats. (**B**) Forward repeats, reverse repeats, palindromic repeats and complementary repeats among all the species of *Solanales*. The *x*-axis represents the name of species, and *y*-axis represents the number of repeats.

The majority of angiosperm possess different forms of repeats in their mitogenome. Microsatellites or SSRs are short stretches of nucleotide motifs that repeatedly occur in the mitogenome. They have been widely used for species identification, genetic diversity studies, and evolutionary analysis, due to their high polymorphism level among the related species [68,69]. A total of 29 repeats were present in the mitogenome of *E. alsinoides*, including 21 mono-, 4 di-, and 4 tri- repeats. The mono-repeat A/T was highly predominant in the mitogenome, compared to the other types of repeats. The size of the repeats was in the range of 5–14 base pairs. SSR comparison between the mitogenome of *E. alsinoides* and nine species of the order *Solanales* showed that *S.lycopersicum* and *P.orientalis* have the highest repeats. *E. alsinoides* have the least number of repeats among all the species, compared to *S.lycopersicum* and *P.orientalis* (Figure 2A). The length of repeats in all the species of *Solanales* range from 5 bp to 22 bp. The species *P.orientalis* shows the largest repeat size, and *I. nil* has the smallest size. Only mono-, di- and tri- repeats are present in all the species of the order *Solanales* (Supplementary Table S5). The A/T mono-repeats are abundantly present in all the mitogenome of *Solanales*. Among the order *Solanales*, the number of SSRs are comparatively less than other angiosperms, and the repeat size is also less. In addition to the SSRs, a total of 199 repeats were identified in the mitogenome of *E. alsinoides*, which include forward repeats (49), reverse repeat (50), palindromic repeat (50), and complementary repeats (50) (Figure 2B). Based on the comparative analysis among the species of *Solanales*, the total number of repeats present in all the species range from 196 to 200 (Supplementary Table S6). The variation among forward, reverse, palindromic and complementary repeats among the species of *Solanales* are represented in Figure 2B. The size of repeats is almost similar in *N. attenuate*, *S. pennellii* and *E. alsinoides*. The size of forward and palindromic repeats is larger in all the species of *Solanales*. The size of the repeats ranges from 15 bp to 9300 bp. The size of the forward and palindromic repeats was >100 in all the species.

### 3.6. RNA Editing Site Prediction

**Figure 3:**
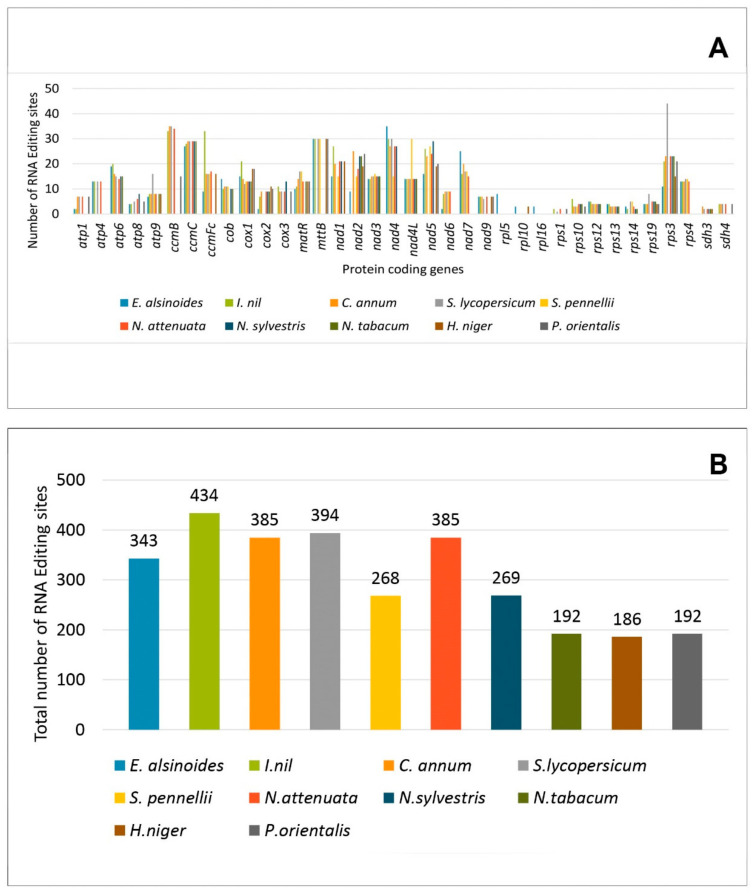
RNA editing sites of all the species. (**A**) RNA editing sites are present in the PCGs. The *x*-axis represents the PCGs in all the species, and *y*-axis represents the number of RNA editing sites. (**B**) Total number of RNA editing sites present in each species. The *x*-axis represents the number of species, and *y*-axis represents the total number of RNA editing sites.

RNA editing is necessary for gene expression in the mitochondrial and chloroplast genomes of all the angiosperms [70,71,72,73]. A total of 343 RNA editing sites have been identified in the 31 PCGs of the *E. alsinoides* mitogenome. Among the 343 RNA editing sites, 27.7% (102) were present in the first position of the codon, and 70.3% (368) were present in the second position, and none of the RNA editing sites were present in the third position of the codon. Most RNA editing sites in the third position did not alter the amino acid encoded by the codon. In the selected species of *Solanales* used for present study, all the RNA editing sites are cytidine (C) to uridine (U) transitions. Cytidine (C) to uridine (U) transitions were found in the RNA editing sites of the mitogenome [74]. The most frequently used amino acid replacement that occurred in the RNA editing site of *E. alsinoides* appeared to be leucine. RNA editing sites have been reported in all PCGs except *sdh3*. The PCGs *nad4*, *ccmC*, and *mttB* (27–35 substitutions) have the higher number of RNA editing sites, while *atp1*, *nad6*, *cox2* (2 substitutions) genes have a lesser number of RNA editing sites (Figure 3A). When comparing the RNA editing sites of all the *Solanales*, the *rps3* gene (44 substitutions) of *S. lycopersicum* showed the highest number of RNA editing sites. The *ccmB* gene of *I. nil*, *C. annum*, *S. lycopersicum*, and *N. attenuate* have 32–35 substitutions for RNA editing sites (Supplementary Table S7). The *ccmC* and *nad4* genes of *E. alsinoides, I. nil, C. annum, S. lycopersicum, Nicotiana attenuata*, and *Nicotiana sylvestris* have the highest number of (27–35) substitutions for RNA editing sites. The *mttB* gene has 30 substitutions for *E. alsinoides, I. nil, S. lycopersicum, S. pennellii, H. niger*, and *P. orientalis*. The RNA editing sites cause an alteration in the initiation and termination of the codons. The RNA editing sites that are present in some PCGs of *Solanales* are conserved, except a few genes such as the ribosomal proteins *rpl* and *rsp*. Based on the comparison, 434 RNA editing sites were found in *I. nil*, which encodes the most RNA editing sites, while *H. niger* (186) has the minimum number of RNA editing sites (Figure 3B). The number of RNA editing sites varies among all the species of *Solanales*. The PCGs in the *E. alsinoides rps10*, *rps3*, and *nad2* use ACG as their initiation codons, but this changes to AUG during RNA editing. The same RNA modification occurs in various PCGs among all the species of *Solanales* that are considered for the present study.

### 3.7. Synonymous and Nonsynonymous Substitution Ratio

**Figure 4:**
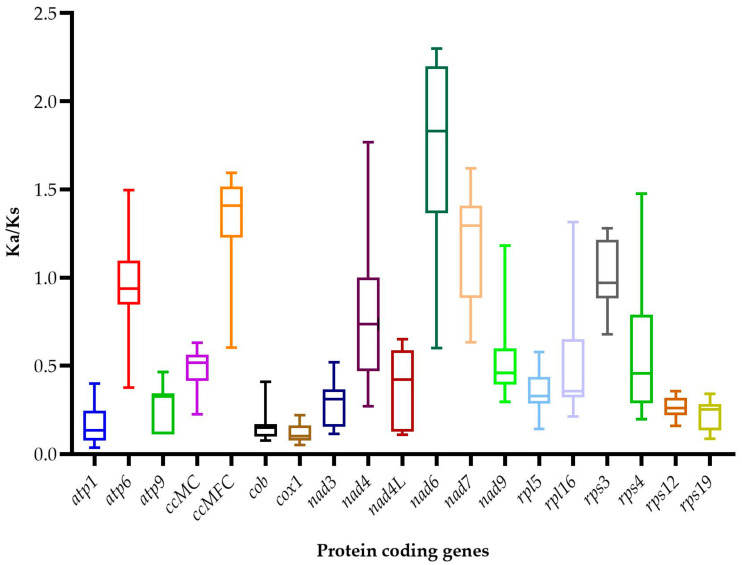
Box plot for pairwise divergence nonsynonymous and synonymous ratio (Ka/Ks) for 19 shared PCGs of all the *Solanales* mitogenomes.

The ratio of nonsynonymous and synonymous substitutions (Ka/Ks) were calculated in order to detect the selection pressure among 19 shared PCGs in the mitogenome of *E. alsinoides* and the species of the order *Solanales*. The selective pressure on PCGs evolution is evaluated by comparing the rate of nonsynonymous (Ka) and synonymous nucleotide substitutions (Ks) [75,76]. The Ka/Ks ratio was calculated to detect the selection pressure among 19 shared PCGs in the mitogenome of *E. alsinoides* and the species of the order *Solanales*. The Ka/Ks ratio >1 shows that the nonsynonymous substitutions exceed synonymous substitutions, which implies that the sequences are less conserved throughout evolution [77]. Here, among the 19 genes, 8 genes (*atp6, nad6, ccmFC, nad4, rpl16, rps3, rps4, nad7*) have a Ka/Ks value of >1 (Figure 4), and they were under positive selection, suggesting that some selective advantage had emerged during evolution, and they were fixed in the population [78]. Eleven genes (*atp1, ccmC, cob, cox1, rps19, rps12, nad3, nad9, atp9, rpl5, nad4L*) have a Ka/Ks ratio <1; this indicates that the synonymous substitutions were tolerated compared to nonsynonymous substitution, implying that the sequences are highly conserved. The results showed that the average Ka/Ks ratio ranges from 0.102 in *cox1* to 0.516 in *ccmC*. The order of the genes are as follows: *cox1* < *atp1* < *cob* < *rps19* < *rps12* < *nad3* < *atp9* < *rpl5* < *nad4L* < *nad9* < *ccmC*. These genes were well conserved and were under purifying selection. When Ka/Ks is equal to one, the number of synonymous and nonsynonymous substitutions are equal [77]. One-way ANOVA (Supplementary Table S4) was performed in order to evaluate the statistical significance (*p* < 0.05) of negative selection, using rps3 as the control group, since most of the mean values fall near one. The result showed that all the genes with a Ka/Ks ratio of <1 were highly significant, except *nad9*. The lowest Ka/Ks ratio was for the *cox1* gene, which signifies lesser amino acid changes; hence, it could be used as a molecular marker for species identification and phylogenetic studies. Several studies have explored the potential of the *cox1* gene used as a molecular marker in plants [79,80].

### 3.8. Codon Usage Analysis

**Figure 5:**
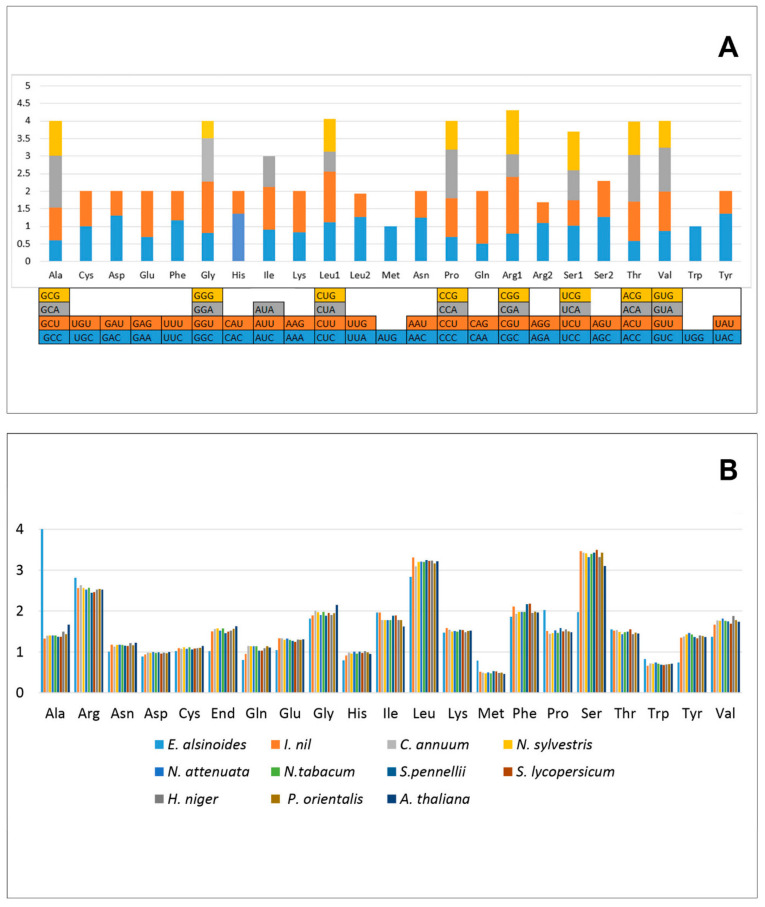
Codon usage of the mitochondrial PCGs. (**A**) Relative synonymous codon usage. Codons are plotted on the *x*-axis and represented in different colors. RSCU values are plotted on *y*-axis. (**B**) Relative synonymous codon usage for all the species. Codons are plotted on the *x*-axis and RSCU values are plotted on *y*-axis.

In the mitogenome of *E. alsinoides*, most PCGs have AUG as the start codon and UAA as the stop codons, with a few exceptions in the PCGs *atp6, ccmF*, and *sdh3* (Figure 5). The codon usage analysis shows that AGA (Arg) and GCU (Ala) are the most frequently used codons, and GGC (Gly) and CAG (Gln) are the least frequently used codons in the PCGs of the mitogenome. The codon usage bias was measured by calculating the relative synonymous codon usage (RSCU) [81]. The relative synonymous codon usage (RSCU) analysis revealed that all the codons are present in the PCGs. A total of 9023 codons were present in 31 unique PCGs of the *E. alsinoides* mitogenome. The RSCU values > 1 indicate high codon usage bias, and the RSCU values < 1 indicate less codon usage bias in the mitogenome PCGs. The RSCU values of the NNU and NNA codons are higher than 1.0, except for two codons, Ile (AUA) and Leu (CUA). The RSCU value of 29 codons in the PGCs was >1, which indicates a strong A or T bias in the third position of the codon in the PCGs of the *E. alsinoides* mitogenomes, which is commonly observed in all the studied plant mitogenomes [82]. The effective number of codons (ENC) was calculated for 31 PCGs of *E. alsinoides*, to understand the codon usage analysis. The ENC values were in the range from 37.6 (*sdh3*) to 60.6 (*atp4*), the majority of which were greater than 40, indicating a weak codon bias in the PCGs of the *E. alsinoides* mitogenome. Comparing *E. alsinoides* with the order *Solanales* revealed that most amino acids are similar in all the species, except a few (Ala, Arg, Gln, Glu, Met, Ser, and Tyr). The percentage of the amino acids Ala, Arg, Met, and Trp was high, whereas the amino acids Gln, Glu, and Tyr were less in the *E. alsinoides* mitogenome than other species among *Solanales*. The presence of the amino acid Met was high in the *E. alsinoides* mitogenome. Met is the start codon for most PGCs; hence, alternate start codons are less than other species of *Solanales*.

### 3.9. Gene Arrangement and Synteny Analysis

**Figure 6:**
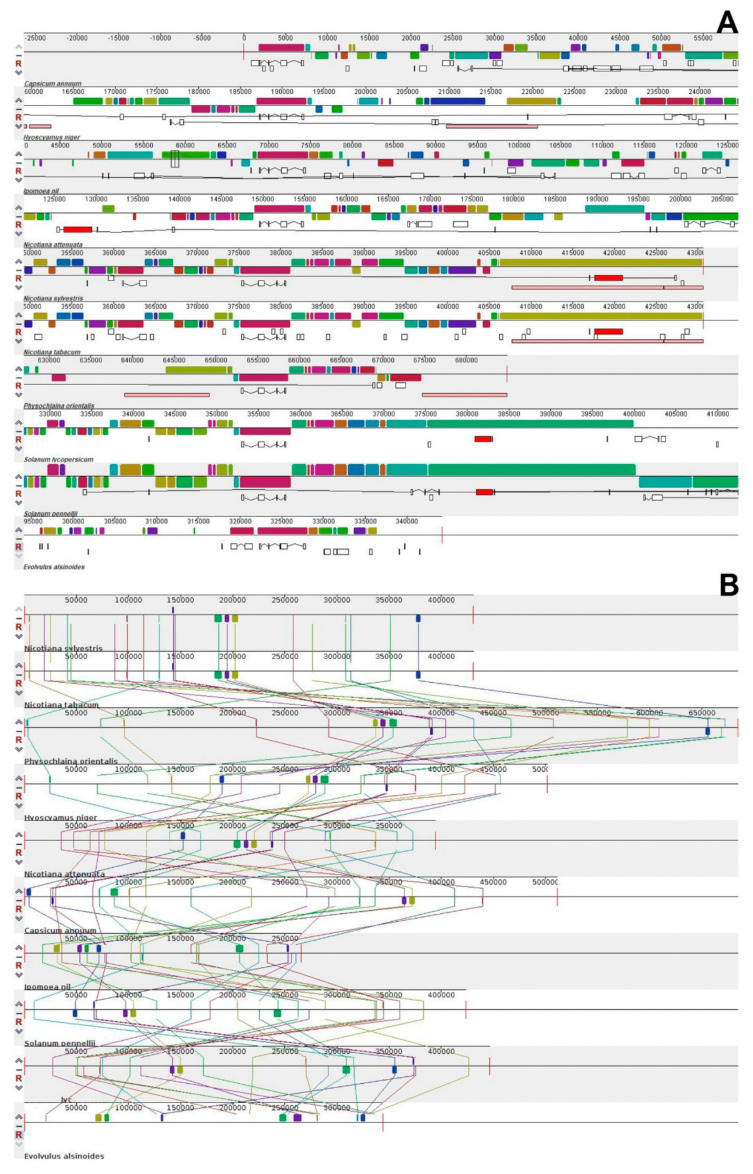
(**A**) Gene order and alignment of genes among all the species of *Solanales*. The genes are represented in colored blocks. (**B**) Nine genes that forms the synteny blocks are represented in colored blocks. The thick-colored box represents the *nad* genes.

The genome size, gene content, and gene order in the mitochondrial genome of higher plants vary significantly, and gene loss frequently occurs during evolution [83]. It is evident from the analysis that the gene arrangement is highly variable in the PCGs and tRNAs of the order *Solanales* (Figure 6A). A small gene block consisting of *nad5, nad4, nad2* was conserved in a few species of *Solanales* (Figure 6B). A total of nine synteny blocks, representing nine genes (*nad9, nad2, ccmFc, nad1, nad4, nad5, matR, cox1, nad7*), were observed in all the species. The two species *N. sylvestris* and *N. tabacum* have the same number of genes, and the gene arrangement is also similar in both the species. The gene blocks *nad5, nad4*, and *nad2* were present in the same gene arrangement in all the three *Nicotiana* species (*N. sylvestris*, *N. tabacum, N. attenuate*). *H. niger, C. annum*, and *P. orientalis* show the presence of these gene blocks with gene rearrangement. The position and arrangement of genes varies among all the species that were considered for the study (Supplementary Figure S1). The species that belong to the same family, which are evolutionary related, show the same gene arrangement in their mitogenome [84]. In all other species, such as *S. pennellii, S. lycopersicum*, *I. nil* and *E. alsinoides*, there are only *nad5* and *nad4* gene blocks with gene rearrangement. The *nad2* gene is present in some other locations in the mitogenome. The species under the *Solanales* were not closely related, except *N. sylvestris* and *N. tabacum*, hence it could be concluded that a high level of gene rearrangement has occurred in all the species.

The pairwise comparison of the mitochondrial gene order in *Solanales* shows that *N. sylvestris*, *N. tabacum, N. attenuate*, and *H. niger* are related, and come under a single group. *C. annuum, P. orientalis* and *I. nil* together form another group. The next group of species consisted of *E. alsinoides, S. lycopersicum* and *S. pennellii*, which form a single entity (Supplementary Table S8). These results were almost similar by their position in the phylogenetic tree. The gene arrangement shows that the genes *nad4, cox1*, and *nad7* are present as blocks, and *rpl5* and *rps12* are present in reverse transposition, but at different locations in the genome of *E. alsinoides* and *S. lycopersicum*. The genes *cox1, nad7, ccmC*, and *atp6* are present as a block of two genes, but there was transposition of these genes in *E. alsinoides* and *S. pennellii*.

### 3.10. Phylogenetic Analysis

**Figure 7:**
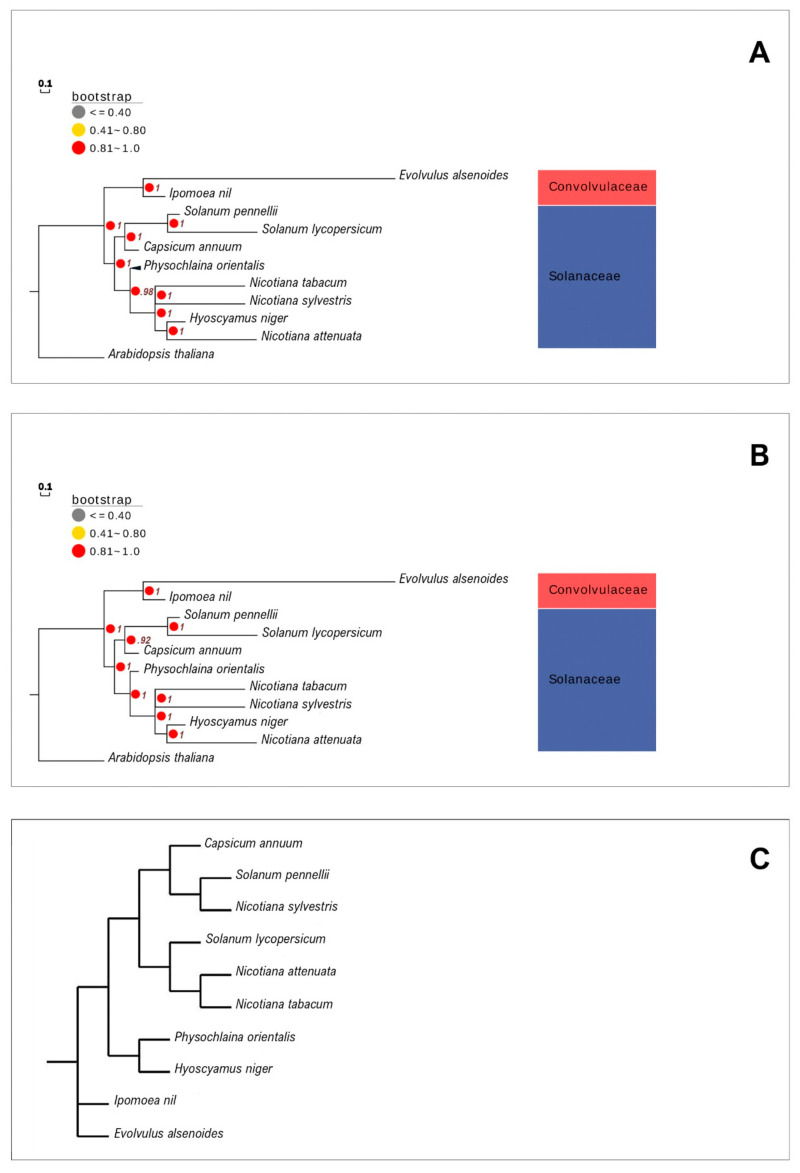
Original phylogenetic tree and reconstructed phylogenetic tree. (**A**) Phylogenetic tree based on 19 shared PCGs using maximum likelihood (ML) tree. (**B**) Phylogenetic tree based on 19 shared PCGs using Bayesian inference (BI). The values in the nodes represent bootstrap value. (**C**) Reconstructed phylogenetic tree according to the gene order.

The mitogenome provides valuable information to understand the evolutionary position of the species [27,49,50]. The results of the phylogenetic analysis showed that both the ML and BI trees were highly similar in their tree topology, in all the constructed partitioned trees. Phylogenetic trees formed two clades (Figure 7A,B), of which clade I consisted of the species belonging to the family *Convolvulaceae* (*E. alsinoides* and *I. nil)*, and Clade II belonged to the species of the family *Solanaceae*. Clade II is divided into the following two subclades: subclade A, which consists of *S. pennellii*, and *S. lycopersicum. C. annum* forms a separate branch in subclade A, indicating that it has diverged from the genus *Solanum*. Subclade B forms the *Nicotiana* species and *H. niger*, in which *H. niger* is closely related to *N. attenuate. P. orientalis* forms a separate branch in subclade B, as it has diverged from the genus *Nicotiana* and *Hyoscamus. A. thaliana*, the outgroup that forms a separate branch from all the other species, belongs to the distinctly related family *Brassicaceae*. In the present BI analysis, the ESS values were greater than 200, which indicates good convergence and that the predicted tree was well determined. From the phylogenetic analysis, it is evident that *E. alsinoides* is distinctly related to all the other species of *Solanaceae*. It forms a separate clade in the phylogenetic tree with *I.nil*. *E. alsinoides* forms a long branch length in the constructed phylogenetic tree. There was no change in the constructed phylogenetic tree tropology after adopting the methods to avoid long branch lengths. These results reveal that the long branch length of the species of *E. alsinoides* could be due to a lack of taxas in the family *Convolvulaceae*.

The phylogenetic tree was reconstructed using genome rearrangement and gene order data. It also provides an estimate of the ancestral genome. The reconstructed phylogenetic tree forms four clades; clade I and clade II consist of *E. alsinoides* and *I. nil*, which form two separate clades from the ancestor. *H. niger* and *P. orientalis* form clade III. Clade IV consists of *C. annum, Solanum* species, and *Nicotiana* species, divided into two subclades (Figure 7C). From the reconstructed phylogenetic tree, it is evident that highly variable gene orders are present in the species *H. niger, P. orientalis, I. nil, and E. alsinoides. I. nil* and *E. alsinoides* belong to the same family *Convolvulaceae*, but they are not closely related genera. Hence, from the reconstructed phylogenetic tree, we can conclude that *E. alsinoides* belong to a separate group from the *Solanales*, and the gene order is also highly variable.

## 4. Conclusions

In summary, *E. alsinoides* is the first sequenced genome in the family Convolvulaceae. So far, only a few mitogenomes are available from the order *Solanales*. The *E. alsinoides* mitochondrial genome and its detailed analysis will provide a base for future investigations on angiosperms, specifically for the family *Convolvulaceae*. The complete mitogenome information that is presented in the present study would serve as a valuable genetic resource for further studies on population genetics, species identification, gene arrangement, and evolutionary studies.

## Figures and Tables

**Table 1:** Genes present in the mitochondrial genome of *E. alsinoides*.

Group of Genes	Gene Names
Complex I (NADH dehydrogenase)	nad1, nad2 ^a,c^, nad3, nad4 ^a^, nad4l, nad5 ^a,^ nad6, nad7 ^a,d^, nad9
Complex II (succinate dehydrogenase)	sdh3
Complex III (ubiquinol cytochrome c reductase)	cob
Complex IV (cytochrome c oxidase)	cox1 ^a^, cox2 ^b(2)^
Complex V (ATP synthase)	atp1, atp4, atp6, atp8, atp9 ^b(2)^
Cytochrome c biogenesis	ccmc, ccmfc^a^
Ribosomal proteins (SSU)	rps3 ^a^, rps4 ^b(2)^, rps12,rps13, rps14,rps19
Ribosomal proteins (LSU)	rpl5, rpl10 ^b(2)^, rpl16
Maturases	matr
Transport membrane protein	mttB
Ribosomal RNAs	rrn26 ^b(2)^, rrnS ^b(3)^, rrnL^b^
Transfer RNAs	trnC ^a^, trnG ^a^, trnH ^b(4^), trnL ^b(3)^ trnM ^b(8)^, trnN, trnP, trnR ^b(2)^, trnS, trnV ^b(2)^, trnW ^a^, trnY

^a^—intron-containing genes, ^b^—duplicated copies of the genes, number of duplicates are represented in the bracket, ^c^—gene containing two introns, ^d^—gene containing three introns. Apex notation indicates the number of copies.

**Table 2:** Genome comparison of *Solanales*.

Species Name	Genome Size	Total Gene Content	No of PCGs	rRNA	tRNA	AT%	GC%	AT Skew	GC Skew
*E. alsinoides*	344184	67	35	6	26	56.46	43.54	0.0016	0.0018
*I. nil*	265768	53	30	3	20	55.55	44.45	0.0075	−0.0012
*N. tabaccum*	430597	62	35	4	23	55.04	44.96	0.0072	0.0057
*N. attenuata*	394341	68	40	4	24	55.01	45.05	0.0020	0.0086
*N. sylverstris*	430597	64	37	4	23	55.04	44.96	0.0072	0.0057
*S. lycopersicum*	446257	41	39	5	27	54.93	45.07	0.0025	−0.0005
*S.pennellii*	423596	70	41	5	24	55.01	44.99	−0.0059	−0.0050
*C. annum*	511530	61	33	3	25	55.48	44.52	0.0069	0.0019
*H. niger*	501401	70	38	4	28	54.82	45.18	−0.0015	0.0003
*P. orientalis*	684857	65	37	4	24	55.19	44.81	−0.0001	−0.8216

## Data Availability

Not applicable.

## Supplementary Materials

The following are available online at https://www.mdpi.com/article/10.3390/life11080769/s1, Table S1: Assembly statistics of mitogenome, Table S2: Complete annotation details of mitogenome, Table S3: Details of tRNA, Table S4: List of shared genes and their statistical analysis of Ka/Ks ratio, Table S5: Repeat annotation using MISA, Table S6: Repeat annotation using reputer, Table S7: Prediction of RNA editing sites, Table S8: Gene rearrangement matrix, Table S9: Species details.
