# Supplementary material for: Genome Characterization, Comparison and Phylogenetic Analysis of Complete Mitochondrial Genome of Evolvulus alsinoides Reveals Highly Rearranged Gene Order in Solanales
Source: Life (Basel). 2021 Jul 30;11(8):769. doi: 10.3390/life11080769 (PMC8398076; doi:10.3390/life11080769)
Supplement: Supplementary file 1 [file life-11-00769-s001.zip › life-1278667-supplementary/Supplementary Table 1.pdf]

Assembly statistics of mitogenome

Assembly statistics	Number
# contigs (>= 0 bp)	3578
# contigs (>= 1000 bp)	408
# contigs (>= 5000 bp)	33
# contigs (>= 10000 bp)	15
# contigs (>= 25000 bp)	2
# contigs (>= 50000 bp)	1
Total length(>= 0 bp)	2089438
Total length(>= 1000 bp)	1048459
Total length(>= 5000 bp)	378781
Total length(>= 10000 bp)	251873
Total length(>= 25000 bp)	87438
Total length(>= 50000 bp)	56887
# contigs	678
Largest contig	56887
Total length	1242295
GC (%)	39.91
N50	2363
N75	1219
L50	108
L75	304
# N's per 100 kbp	32.20

Assembly statistics	Number
# contigs (>= 0 bp)	9767
# contigs (>= 1000 bp)	756
# contigs (>= 5000 bp)	90
# contigs (>= 10000 bp)	25
# contigs (>= 25000 bp)	10
# contigs (>= 50000 bp)	1
Total length(>= 0 bp)	4165936
Total length(>= 1000 bp)	2419322
Total length(>= 5000 bp)	1078417
Total length(>= 10000 bp)	669019
Total length(>= 25000 bp)	466149
Total length(>= 50000 bp)	175537
# contigs	1140
Largest contig	175537
Total length	2721611
GC (%)	39.6
N50	3326
N75	1605
L50	160
L75	458
# N's per 100 kbp	14182.78
