# Supplementary material for: Genome Characterization, Comparison and Phylogenetic Analysis of Complete Mitochondrial Genome of Evolvulus alsinoides Reveals Highly Rearranged Gene Order in Solanales
Source: Life (Basel). 2021 Jul 30;11(8):769. doi: 10.3390/life11080769 (PMC8398076; doi:10.3390/life11080769)
Supplement: Supplementary file 1 [file life-11-00769-s001.zip › life-1278667-supplementary/Supplementary Table 4.pdf]

<u>List of Shared Genes</u>

GENES	*Evolvulus alsinoides*	*Ipomoea nil*	*Capsicum annuum*	*Nicotiana attenuata*	*Arabidopsis thaliana*	*Nicotiana sylvestris*	*Nicotiana tabacum*	*Solanum pennellii*	*Solanum ly copersicum*	*Physochlaina orientalis*	*Hyoscyamus niger*
nad1											
nad2											
nad3											
nad4											
nad4L											
nad5											
nad6											
nad7											
nad9											
sdh3											
sdh4											
cob											
cox1											
cox2											
cox3											
atp1											
atp4											
atp6											
atp8											
atp9											
ccmB											
ccmC											
ccmFC											
ccmFN											
matR											
mttB											
rpl2											
rpl5											
rpl10											
rpl16											

rps1											
rps3											
rps4											
rps10											
rps12											
rps13											
rps14											
rps19											

Legend
Green Colour: Represent shared genes among all species of the order Solanales
Red Colour: Genes not present in the species

<u>One-way ANOVA (Ka/Ks) ratio</u>

Number of families	1					
Number of comparisons per family	18					
Alpha	0.05					
Dunn's multiple comparisons test	Mean rank diff.	Significant ?	Summary	Adjusted P Value	P-?	
rps3 vs. *atp1*	390.3	Yes	****	<0.0001	A	*atp1*
rps3 vs. *atp6*	21.02	No	ns	>0.9999	B	*atp6*
rps3 vs. *atp9*	302.9	Yes	****	<0.0001	C	*atp9*
rps3 vs. *ccMC*	161.6	Yes	**	0.0047	D	*ccMC*
rps3 vs. *ccMFC*	-70.06	No	ns	>0.9999	E	*ccMFC*
rps3 vs. *cob*	392.5	Yes	****	<0.0001	F	*cob*
rps3 vs. *cox1*	436.6	Yes	****	<0.0001	G	*cox1*
rps3 vs. *nad3*	277	Yes	****	<0.0001	H	*nad3*
rps3 vs. *nad4*	81.28	No	ns	0.635	I	*nad4*
rps3 vs. *nad4L*	252	Yes	****	<0.0001	J	*nad4L*
rps3 vs. *nad6*	-87.05	No	ns	>0.9999	K	*nad6*
rps3 vs. *nad7*	-37.12	No	ns	>0.9999	L	*nad7*
rps3 vs. *nad9*	160.2	No	ns	0.0612	M	*nad9*
rps3 vs. *rpl5*	236.9	Yes	****	<0.0001	N	*rpl5*
rps3 vs. *rpl16*	178.6	Yes	***	0.0002	O	*rpl16*
rps3 vs. *rps4*	153.9	Yes	**	0.0012	Q	*rps4*
rps3 vs. *rps12*	311.8	Yes	****	<0.0001	R	*rps12*

rps3 vs. *rps19*	348	Yes	****	<0.0001	S	*rps19*
Test details	Mean rank 1	Mean rank 2	Mean rank diff.	n1	n2	
rps3 vs. *atp1*	496.8	106.5	390.3	38	38	
rps3 vs. *atp6*	496.8	475.8	21.02	38	52	
rps3 vs. *atp9*	496.8	193.9	302.9	38	23	
rps3 vs. *ccMC*	496.8	335.3	161.6	38	29	
rps3 vs. *ccMFC*	496.8	566.9	-70.06	38	32	
rps3 vs. *cob*	496.8	104.4	392.5	38	34	
rps3 vs. *cox1*	496.8	60.24	436.6	38	37	
rps3 vs. *nad3*	496.8	219.8	277	38	32	
rps3 vs. *nad4*	496.8	415.6	81.28	38	50	
rps3 vs. *nad4L*	496.8	244.8	252	38	26	
rps3 vs. *nad6*	496.8	583.9	-87.05	38	9	
rps3 vs. *nad7*	496.8	534	-37.12	38	27	
rps3 vs. *nad9*	496.8	336.6	160.2	38	15	
rps3 vs. *rpl5*	496.8	260	236.9	38	43	
rps3 vs. *rpl16*	496.8	318.3	178.6	38	39	
rps3 vs. *rps4*	496.8	342.9	153.9	38	50	
rps3 vs. *rps12*	496.8	185.1	311.8	38	20	
rps3 vs. *rps19*	496.8	148.9	348	38	27	
