# Supplementary material for: Genome Characterization, Comparison and Phylogenetic Analysis of Complete Mitochondrial Genome of Evolvulus alsinoides Reveals Highly Rearranged Gene Order in Solanales
Source: Life (Basel). 2021 Jul 30;11(8):769. doi: 10.3390/life11080769 (PMC8398076; doi:10.3390/life11080769)
Supplement: Supplementary file 1 [file life-11-00769-s001.zip › life-1278667-supplementary/Supplementary Table 8.pdf]

Species	Ea	ca	Hn	In	Na	Ns	Po	Sl	Sp
Evolvulus alsinoides	234	20	18	18	40	24	18	12	30
capsiccum annum	20	300	24	20	20	14	16	10	8
Hyoscyamus niger	**18**	24	266	14	10	28	18	6	12
Ipomoea nil	**14**	**18**	**12**	300	**10**	**8**	**8**	**4**	**2**
Nicotiana attenuata	40	20	10	10	266	**22**	12	38	20
Nicotiana sylvestris	24	14	28	8	22	266	12	22	14
Physochlaina orientalis	18	16	18	10	12	12	300	18	28
Solanum lycopersicum	12	10	6	4	38	22	18	176	16
Solanum pennellii	30	8	12	4	20	14	28	16	204

**Nicotiana sylvestris* and *Nicotiana tabacum* have the same genomic features so they have considered only one entity for the analysis to avoid duplications.
